# Optimizing Photobiomodulation for Smooth Muscle Differentiation of Adipose-Derived Stem Cells Using Retinoic Acid and TGFβ in a Two-Dimensional Model

**DOI:** 10.3390/cells15090789

**Published:** 2026-04-27

**Authors:** Christevie Mbuyu, Heidi Abrahamse, Anine Crous

**Affiliations:** Laser Research Centre, Faculty of Health Sciences, University of Johannesburg, Doornfontein 2028, South Africa; christeviem@uj.ac.za (C.M.); habrahamse@uj.ac.za (H.A.)

**Keywords:** photobiomodulation, smooth muscle differentiation, adipose-derived stem cells, regenerative medicine

## Abstract

**Highlights:**

**What are the main findings?**

- Photobiomodulation (PBM) modulated adipose-derived stem cell behaviour during smooth muscle (SM) differentiation in a fluence-dependent biphasic manner, where 5 J/cm^2^ enhanced proliferation, mitochondrial activity, extracellular matrix production and smooth muscle marker expression.
- Higher PBM fluence (10 J/cm^2^) suppressed metabolic activity and promoted increased migration, indicating delayed differentiation and a more synthetic-like SM phenotype compared with lower-fluence conditions.

**What are the implications of the main findings?**

- Green light PBM (525 nm) at 5 J/cm^2^ emerged as the most favourable parameter for promoting ADSCs progression toward a contractile-like SM phenotype.
- These findings highlight PBM as a promising non-invasive approach to optimize stem-cell differentiation for smooth muscle tissue engineering and regenerative medicine applications.

**Abstract:**

Smooth muscle (SM) dysfunction contributes to several pathological conditions, including atherosclerosis; current treatment strategies often fail to restore functional contractility. Adipose-derived stem cells (ADSCs) offer a promising cell source for regenerative medicine due to their accessibility and multipotency. Their differentiation into smooth muscle cells (SMC) is commonly driven by biochemical cues such as retinoic acid and transforming growth factor β; however, supporting this process with additional, non-invasive stimuli may enhance outcomes. Photobiomodulation (PBM) has emerged as a potential modulator of cellular metabolism, mitochondrial function and lineage commitment; however, its role in ADSCs to SMC differentiation remains insufficiently defined. ADSCs were irradiated with green (525 nm), near-infrared (825 nm) or dual wavelengths at 5 J/cm^2^ and 10 J/cm^2^ alongside the growth factors. Proliferation, cytotoxicity, mitochondrial membrane potential, collagen production, migration and smooth muscle marker expression were assessed. PBM induced a fluence-dependent biphasic response. 5 J/cm^2^ fluences enhanced proliferation, mitochondrial activity, collagen deposition and organized SMC marker expression, whereas 10 J/cm^2^ fluences lowered proliferation and membrane potential, reduced collagen and increased migration. PBM at 5 J/cm^2^, especially greenlight, most effectively promoted ADSCs’ progression towards a SMC-like phenotype, with features consistent with a more contractile-like state.

## 1. Introduction

Smooth muscle dysfunction refers to the impaired ability of SM tissues to perform their essential contractile and relaxation functions, leading to various health concerns depending on the affected organ systems [1,2,3]. These muscles, located in many hollow organs, are crucial for the maintenance of normal physiological processes [1,2,3]. Among the numerous diseases affecting SM, atherosclerosis is a chronic, systemic inflammatory condition targeting medium and large arteries, including the coronary, carotid, peripheral arteries, and the aorta [4,5]. It remains a significant global health problem and contributes substantially to the current burden of cardiovascular diseases worldwide.

Atherosclerosis is characterized by the accumulation and consequent thickening of the arterial walls by lipids, inflammatory cells and SMC, leading to plaques. It is a major cause of morbidity and mortality globally [4,5,6]. Its prevalence increases with age, with men being more affected than women [7]. Alarmingly, the burden of atherosclerosis-related disease shows no signs of decline but rather is trending upwards in the upcoming years, highlighting the urgent need for effective therapeutic solutions. Among emerging approaches, regenerative therapy is currently being explored as a promising potential treatment for degenerative diseases or injuries affecting the SM [8]. For atherosclerosis in particular, regenerative therapies involving the use of mesenchymal stem cells are being investigated as treatments in the modulation and repair of atherosclerotic plaques [9]. These therapies aim to promote regeneration of damaged cells in organs or tissues resulting from atherosclerosis. More research is required to establish their efficacy and safety in clinical settings [10].

Photobiomodulation (PBM), previously known as low-level laser therapy, makes use of non-ionizing light sources within the red to near-infrared (NIR) spectrum to modulate cellular activity without causing thermal damage [11]. This non-invasive technique has been demonstrated to enhance the proliferation and differentiation of various stem cells, including adipose-derived stem cells (ADSCs [12]. PBM interacts with some cellular chromophores, such as Cytochrome C oxidase (CCO) and opsins, which prompt the action of various signalling pathways that can promote numerous therapeutic options [13]. It has been proven to be effective in the treatment of multiple conditions, including wound healing, reduction in inflammation and regulation of cognitive processes [11,14,15], making it a reliable option for regenerative medicine and tissue engineering.

ADSCs, which are mesenchymal stem cells derived from adipose tissue with mesenchymal lineage differentiation potential and remarkable possibilities in regenerative medicine [16]. On this note, previous studies have suggested their applicability in regenerative medicine for SM related conditions [17]. SMCs play a vital role in the maintenance of both structural and functional integrity of blood vessels and hollow organs such as the bladder, uterus, vasculature and gastrointestinal tract [18]. Research has shown the ability of ADSCs to differentiate into SMCs, evidenced by the increased expression of SMC markers [19].

Previously, Wang and colleagues (2009) have conducted studies where ADSCs have been shown to differentiate into SMC under biochemical stimulation, particularly in response to transforming growth factor β (TGF-β) and bone morphogenetic proteins, which work by inducing the upregulation of several contractile markers, hence promoting a contractile phenotype. Retinoic acid (RA) has also been reported to modulate SM differentiation [20]. In addition, PBM has been reported in research conducted by Mvula and colleagues (2016) and De Villiers (2010), respectively, to influence stem cell behaviour and enhance differentiation towards a SMC-like phenotype; however, this was achieved with the aid of growth factors: TGF-β or RA, consistently used independently [21,22]. The aforementioned factors are crucial for the differentiation process of ADSCs into SMC; their presence when used in combination with PBM has been demonstrated to increase SMC gene expression [23]. The fundamental basis of differentiation of SMC comprises an interactive process influenced by diverse environmental cues, molecular mechanisms and the activation of key signalling pathways [24,25]. Other works have looked into the in vivo differentiation of ADSC (human) into SMC with the aid of growth factors, however, without the influence of PBM [26]. Despite these advances, studies evaluating the combined effects of TGF-β and RA alongside varying PBM wavelengths and fluences on ADSC-to SMC differentiation remain limited. Therefore, the present study aims to optimize PBM parameters and the influence of combined biochemical (TGF-β and RA) on ADSC differentiation into SMC in a two-dimensional setting over defined time points.

This study highlights the potential of PBM as a tool to enhance SM tissue engineering strategies. Considering the importance of SM in physiological function, a reliable method for directing ADSCs differentiation into SMC could enable a sustainable and functional protocol for potential use in regenerative medicine. The use of growth factors stimulates and guides stem cells to proliferate and differentiate into various cell lines under standard differentiation conditions; moreover, PBM further enhances these processes [27]. Lastly, the study could potentially be beneficial in clinical applications by standardizing PBM parameters. This could enhance the effectiveness and consistency of methods for reproducible outcomes in vitro for successful in vivo treatment translation for SM related disorders [28].

## 2. Materials and Methods

### 2.1. Cell Culture

Immortalized adipose derived stem cells with hTERT ASC52telo (ATCC^®^ SCRC-4000™) were cultured in induction media comprising Dulbecco’s Modified Eagle Media (DMEM) (Sigma-Aldrich, St. Louis, MO, USA; D5796) and supplemented with 10% fetal bovine serum (FBS) (Biochrom, Berlin, Germany; S0615), antibiotics: 0.1% Penicillin-Streptomycin (Sigma-Aldrich, St. Louis, MO, USA; P4333) and 1 µg/mL Amphotericin B (Sigma-Aldrich, St. Louis, MO, USA; A2942), each at 0.5%. The cells were then incubated at 37 °C in a 5% carbon dioxide environment and 85% humidity (Heracell™ 150i CO_2_ Incubator, Thermo Scientific™, Waltham, MA, USA; 51026280) in Corning^®^ cell culture flasks (Corning, NY, USA; CLS431080). Upon reaching semiconfluency (60–80%), the cells were seeded into 96-well plates (NunclonTM Delta Surface, Thermo Fisher Scientific, Waltham, MA, USA). This was achieved by dispensing 10 µL of the prepared cell suspension into each well, ensuring even distribution. The seeded plates were then incubated at 37 °C in 5% CO_2_ and 85% humidity to facilitate cell attachment. A few hours after cell adhesion, the complete media with 10% FBS was removed and replaced with 200 µL of either complete media with reduced 1% FBS or smooth muscle cell induction media (SMIM), depending on the experimental group. SMIM consists of induction media (with 1% FBS) supplemented with growth factors TGF-β (1 ng/mL) (Invitrogen, Thermo Fisher Scientific, Waltham, MA, USA; PHG 9204) and RA (Sigma-Aldrich, St. Louis, MO, USA; R2605) at a concentration of 0.1 µM, used simultaneously–a novel approach. All groups were subjected to media change after every two days for the duration of the treatment period.

### 2.2. Photobiomodulation

Following the seeding of ADSCs and in the 96-well plates at a density of 1 × 10^3^ cells per well, they were allowed to incubate and continue to attach for a period of 24 h prior to irradiation. Irradiation was conducted using Green (G) 525 nm wavelength Diode Laser (National Laser Centre of South Africa, Pretoria, South Africa; EN 60825-1:2007) and Near-Infra Red (NIR) 825 nm Diode Laser (National Laser Centre of South Africa, Pretoria, South Africa; SN 101080908ADR-1800) as well as a combination of both (825 nm and 525 nm), each at a fluence of 5 J/cm^2^ and 10 J/cm^2^. For the combined-wavelength condition, cells were exposed sequentially to 525 nm and 825 nm irradiation within the same session, with each wavelength delivering the specified fluence independently. The FieldMate Laser Power Metre (Coherent, Santa Clara, CA, USA; 1098297) was used to measure the power of laser output (mW). Finally, laser exposure time was calculated based on fluence using a High-Sensitivity Thermophile Sensor PM3 (Coherent, Santa Clara, CA, USA; 1098336). The laser parameters employed are shown in Table 1. PBM was performed in the dark at room temperature (RT) and in line with the experimental parameters stated in the table. Exposure time was determined according to the following formula:

$${mW/cm}^{2}=\frac{mW}{\pi x(r^{2})}$$

$${W/cm}^{2}=\frac{mW{/cm}^{2}}{1000}$$

$$Time(s)=\frac{J{/cm}^{2}}{{W/cm}^{2}}$$

### 2.3. Experimental Design

**Figure 1:**
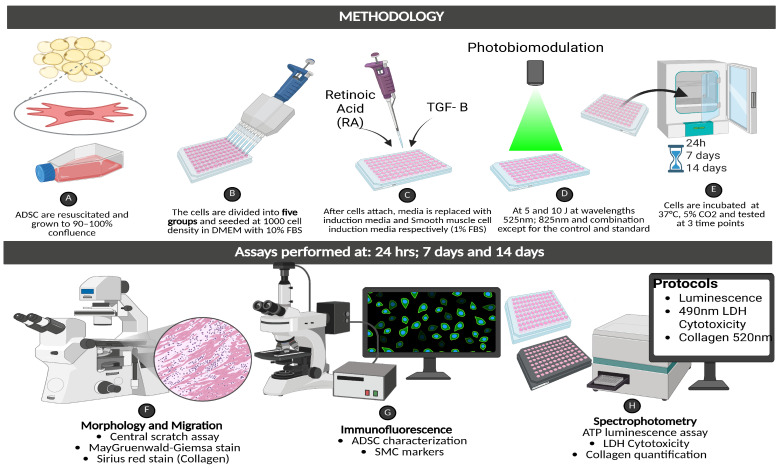
Methodology of ADSCs differentiation into SMC. Created in Biorender. Christevie Mbuyu. (2025).

The experiment comprises five experimental groups: Group 1, Control, which includes ADSCs cultured in SMIM (1% FBS) with no PBM exposure; Group 2, the standard group comprising ADSCs cultured and maintained in complete media with reduced serum (1% FBS) and no PBM exposure. Group 3, ADSCs treated with SMIM irradiated at 525 nm at a fluency of 5 and 10 J/cm^2^ (G 5 J/cm^2^ and G 10 J/cm^2^); Group 4, ADSCs treated with SMIM and exposed to PBM at 825 nm at a fluency of 5 and 10 J/cm^2^ (NIR 5 J/cm^2^ and NIR 10 J/cm^2^); and finally Group 5, ADSCs treated with SMIM exposed to combined PBM wavelengths of 525 and 825 nm at a fluency of 5 and 10 J/cm^2^ (combination 5 J/cm^2^ and 10 J/cm^2^). Post-irradiation cells will be maintained in either complete media with reduced serum or SMIM and assessed at 24 h, 7 days, and then at 14 days to evaluate the differentiation progress. The media was changed every 3 days during this period. The methodology has been represented in Figure 1 below.

### 2.4. Cellular Morphology: Inverted Light Microscopy (Giemsa Stain)

A May-Grunewald Giemsa stain was performed to assess the morphology and evaluate the differentiation of ADSCs into SMCs. 1 × phosphate-buffered Saline (PBS) was used to rinse cells, and thereafter they were fixed in methanol for 5 min. The cells were rinsed again following fixation in PBS and stained for 4 min with May-Grunewald stain and thereafter in Giemsa stain for 6 min before undergoing rinsing thrice in PBS. The morphology of both irradiated and non-irradiated groups was evaluated at 24 h, 7 days and 14 days after PBM exposure using the inverted light microscope (Olympus, Tokyo, Japan CKX41). A microscope-connected digital camera (Olympus, Tokyo, Japan; SC30) was used to capture the images via the getIT programme. Moreover, morphological variations associated with SMC differentiation progress during the 14-day period were also monitored.

### 2.5. Biochemical Analysis

#### 2.5.1. Cell Viability and Proliferation

##### Proliferation and Viability: Adenosine Triphosphate (ATP) Luminescence Assay

The Cell Titer-Glo^®^ 2.0 luminescent cell viability assay (Promega, Madison, WI, USA; G9241) was used to measure Cellular ATP. The ATP reagent was introduced into the cells, causing lysis whilst producing a luminescence signal directly proportional to the levels of ATP. A concentration of 1 × 10^3^ cells/100 µL of cell resuspension was prepared. Following the manufacturer’s instructions, the prepared cell suspension was combined in a 1:1 (50 µL) volume with the reagent in a 96-well microplate. Thereafter, the plate was incubated at room temperature on a shaker (Labcon, Petaluma, CA, USA; 3081U) for 2 min to facilitate cellular lysis and then further incubated for another 10 min in a dark room. Luminescence intensity was measured using the VICTOR NIVO Multilabel Plate Counter (PerkinElmer, Waltham, MA, USA; HH3522019094) at 24 h, 7 days and 14 days after PBM exposure, where luminescence was determined and represented as relative light units (RLUs).

##### Mitochondrial Membrane Potential (MMP) Test

ADSCs were cultured in 96-well plates at a cell density of 1 × 10^3^ in complete media and incubated at 37 °C and 5% CO_2_ until adherent. Prior to irradiation, where applicable, cells were maintained in SMIM and complete media. MMP kit (Sigma MAK 159) was used to assess mitochondrial health and cell vitality according to the manufacturer’s instructions. Mitochondria generate a potential across their membranes as a result of the electron transport chain-associated enzymatic activities. When apoptosis occurs, collapse of the MMP coincides with the opening of mitochondrial permeability transition pores, leading to cytochrome C release into the cytosol, indicating depolarization, in turn triggering other downstream events in the apoptotic cascade. Here, JC-10 is a cationic, lipophilic dye that is concentrated and forms reversible red-fluorescent JC-10 aggregates (λ_ex_ = 540/λ_em_ = 590 nm) in cells with polarized mitochondrial membranes. In compromised or apoptotic cells, MMP collapse results in failure to retain the JC-10 in the mitochondria and thus a return to its monomeric green-fluorescent form (λ_ex_ = 490/λ_em_ = 525 nm). Polarized mitochondria therefore display stronger red fluorescence from dye aggregates alongside green fluorescence from the monomeric form, and the ratio of red to green fluorescence is used as an indicator of mitochondrial membrane potential [29]. Assays were performed at all three timepoints, and analysis was performed using the VICTOR NIVO Multilabel Plate Counter (PerkinElmer, Waltham, MA, USA; HH3522019094). Thereafter, the respective red/green dye ratios were calculated.

#### 2.5.2. Cell Cytotoxicity

##### Lactate Dehydrogenase (LDH) Cytotoxicity Assay

The Cytotox 96^®^ (Promega, Madison, WI, USA; G1780), non-radioactive cytotoxicity assay, is important for the measurement of LDH, an essential enzyme for metabolism and a reliable marker of cellular health and tissue damage. Its release is used to evaluate cytotoxicity or cellular viability; increased levels are indicative of membrane damage and cell death (apoptosis). This assay, therefore, is used to measure cytotoxicity through the detection of LDH released from damaged or lysed cells into the surrounding environment. After each irradiation, 500 µL of culture media was removed from each plate. Thereafter, 100 µL of the collected culture medium was combined with an equal volume of reconstituted substrate mix. The plate was wrapped in foil to protect it from light and incubated at room temperature for 30 min. After incubation, 100 µL of stop solution was added, and the absorbance was measured at 490 nm. Optimum LDH release, indicating definite cellular lysis, was achieved by incubating the plate at −80 °C for 30 min and subsequently thawing it for 15 min at 37 °C. Analysis was performed using the VICTOR NIVO Multilabel Plate Counter (PerkinElmer, Waltham, MA, USA; HH3522019094) at 490 nm.

#### 2.5.3. Extracellular Matrix Remodelling

##### Collagen Assay

Cells were rinsed twice in 1X PBS; thereafter, they were fixed in 4% PFA for 15 min. After fixation, they were washed in PBS and stained with 0.1% Sirius red in saturated picric acid for 1 h at room temperature. Excess dye was removed by gently washing the cells with 0.5% acetic acid until the background cleared, after which samples were imaged microscopically to visualize collagen deposition using (Olympus, Tokyo, Japan; CKX41). For quantitative analysis, the bound dye was elucidated by adding 0.1 NaOH in 50% methanol (200 µL per well in a 96-well plate) and incubating for 30 min with gentle shaking. The solution was read spectrophotometrically at 540 nm, and collagen was expressed as absorbance values relative to the control using the VICTOR NIVO Multilabel Plate Counter (PerkinElmer, Waltham, MA, USA; HH3522019094).

#### 2.5.4. Functional Assay

##### Cell Migration: Central Scratch

The ‘central scratch’ method was used in order to assess cellular motility. Cells were cultured in 96-well plates and incubated overnight at 37 °C with 5% CO_2_. Irradiation exposure was conducted according to the experimental groups. Cells were incubated and maintained at 37 °C with 5% CO_2_ for 14 days, with respective SMIM and complete media change every two days. At day 14, a sterile P-200 pipette tip was used to make a central scratch. 200 µL of HBSS was used to wash the cells; following that, 200 µL of SMIM and complete media according to respective groups was added to the wells. An inverted microscope (Olympus, Tokyo, Japan; CKX41) was used to track cell migration at specific focal plane positions. Three distances were measured for each scratch made across the different groups, and the average was recorded with a digital camera (Olympus, Tokyo, Japan; SC30). Images were captured at 0 h, 24 h, 48 h and 72 h post scratch.

#### 2.5.5. Characterization of ADSCs and SMC Markers

Immunofluorescence was conducted on non-irradiated ADSCs for two days in complete media for characterization (CD 44 (PTE 60224-1-IG, Rosemont, IL, USA); CD 90 (MA5-16671, Invitrogen, Thermo Fisher Scientific, Waltham, MA, USA) and CD 105 (PTE 67075-1-IG, Rosemont, IL, USA)) as well as on the respective experimental groups cultured in 96-well plates to determine SMC differentiation with the following markers: SMAα (PTE 14395-1-AP, Rosemont, IL, USA) (1:800); desmin (PTE 16520-1-AP, Rosemont, IL, USA) (1: 400); calponin (PTE 13938-1-AP, Rosemont, IL, USA) (1:400) and SMHHC (PTE 13938-1-AP, Rosemont, IL, USA) (1: 50) at respective time points. Following removal of media, cells were washed thrice in PBS, fixed with 4% paraformaldehyde for 15 min at ambient temperature. Thereafter, cells were washed thrice in cold PBS, then using Triton X-100 (0.5% Triton X-100 constituted in PBS) to permeabilize cells for 5 min at room temperature (only experimental groups). Following this, cells were rinsed in PBS and incubated in 5% BSA in PBS (blocking buffer) for 30 min to 1 h at RT to finalize blocking. Thereafter, diluted primary antibody was added to the 5% BSA and incubated at 4 °C overnight or for 1 h at RT. Cells were washed three times in PBS to remove unbound primary antibodies. Where necessary, cells were once again incubated, and this time with a fluorescent labelled fluorophore-conjugated secondary antibody (Alexa Fluor 647 ThermoFisher A21235 or A21245, Invitrogen, Thermo Fisher Scientific, Waltham, MA, USA) (diluted in blocking buffer) for 1 h in the dark or 4 °C overnight. The secondary antibody was specific to the host species of the primary antibody used. Cells were washed three times in PBS to remove unbound secondary antibody. Additionally, 4′- 6- diamidino-2-phenylindole (DAPI) (1 µg/mL) was used as a counterstain for nuclear structures. The combination of the cells and DAPI was incubated for 10 min and thereafter washed once with PBS. Results were imaged with the Leica Mica Microhub, Leica Microsystems GmbH, Wetzlar, Germany; distributed by Promolab Pty Ltd., South Africa.

### 2.6. Statistical Analysis

The biochemical assays were performed in duplicate, and spectrophotometry studies were conducted using a blank sample derived from the corresponding collected data. Statistical analysis was conducted using GraphPad Prism software (versions 8 and 9). Data are presented as mean ± standard error of the mean (SEM) (*n* = 3). Differences between groups were analyzed using two-way ANOVA. Statistical significances were expressed as *p* < 0.05 (*), *p* < 0.01 (**), *p* < 0.001 (***) and *p* < 0.0001 (****). Unless otherwise stated, statistical comparisons were performed relative to the control group. In addition, intra-group comparisons between fluences (5 J/cm^2^ vs. 10 J/cm^2^ within the same wavelength group) were analyzed separately and are indicated where relevant. Black asterisks (*) denote comparisons with the control group, while coloured asterisk or (#) indicate intra-group fluence comparisons.

## 3. Results

### 3.1. Cellular Morphology: Inverted Light Microscopy

**Figure 2:**
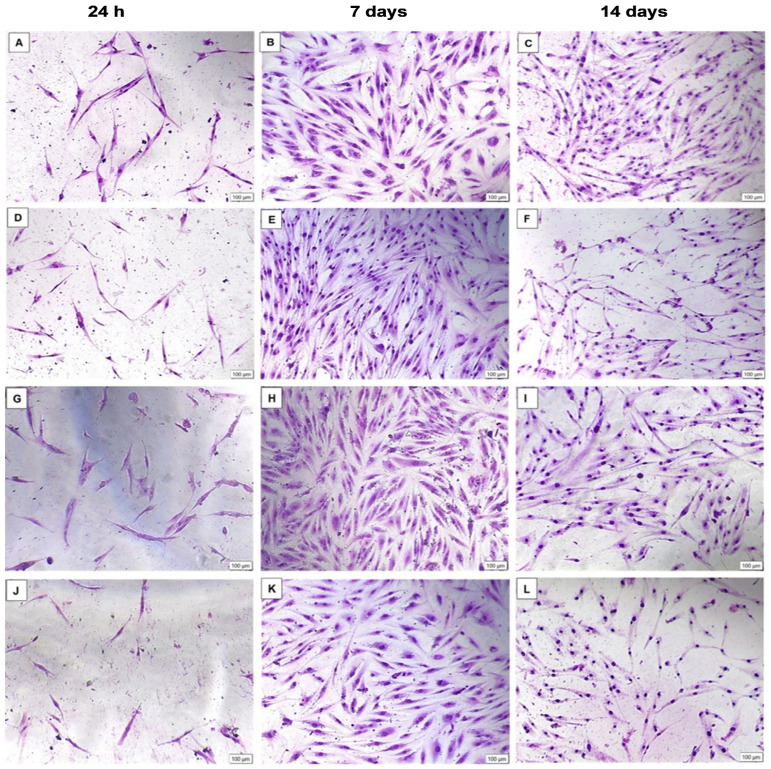

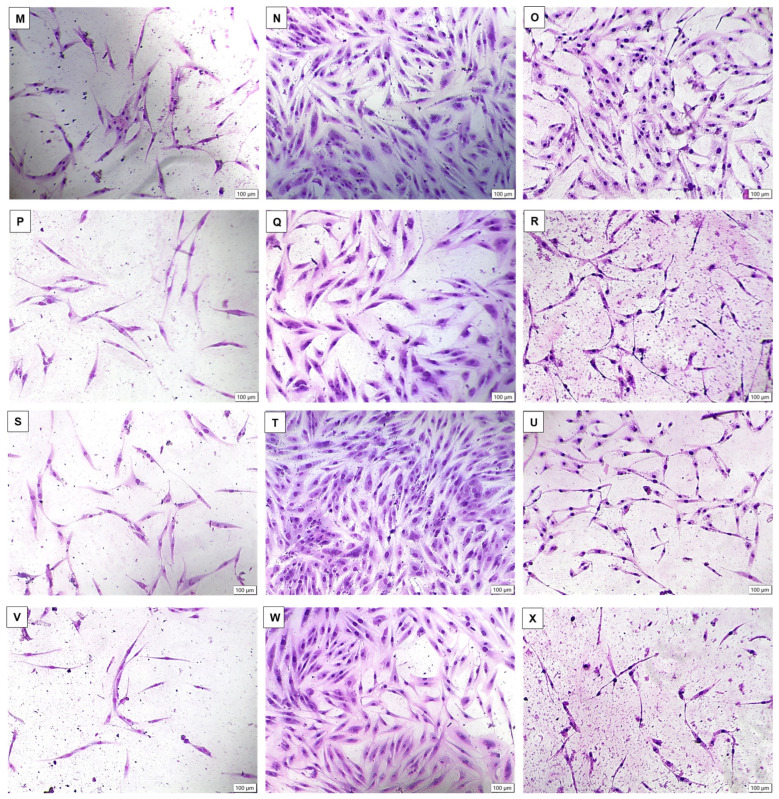
May Grunwald Giemsa-stained morphology of ADSCs at 24 h, 7 days and 14 days. Images (**A**–**C**) = Control, (**D**–**F**) = Standard, (**G**–**I**) = Green 5 J/cm^2^, (**J**–**L**) = Green 10 J/cm^2^, (**M**–**O**) = NIR 5 J/cm^2^, (**P**–**R**) = NIR 10 J/cm^2^, (**S**–**U**) = Combination 5 J/cm^2^, (**V**–**X**) = Combination 10 J/cm^2^. At 24 h, all groups showed uniformly shaped ADSCs. By day 7, proliferation increased in all groups with higher density in the standard and all 5 J/cm^2^ groups, while 10 J/cm^2^ groups were sparser than the control and their 5 J/cm^2^ counterparts. The standard consistently exhibited elongated fibroblast-like morphology. At 14 days, overall cell density declined, but spindle-like SMC morphology was maintained across the PBM and control groups. The standard appeared nutrient-depleted. Among the PBM groups, 5 J/cm^2^ showed less thinning than 10 J/cm^2^, with the combination showing the lowest density.

Morphology results (Figure 2) showed that initially, all groups reflected a homogenous layer of ADSCs with a spindle-like shape, a feat that is characteristic of ADSCs. By day 7, there are no visible morphological distinctions between the control and PBM groups; however, the standard shows a fibroblastic-like morphology with extended ends compared to all other groups. In terms of proliferation, increased proliferation is observed across all groups; however, some groups reflect higher cellular amounts than others, these include the standard and the 5 J/cm^2^ groups of the green, NIR and combination groups. Whilst 10 J/cm^2^ groups of cells were generally more sparse or relative to the control and had a lower cell density compared to their respective 5 J/cm^2^ groups. Finally, at the last time point, it was observed that although proliferation declined, cells maintained their spindle-like form and SMC phenotype with some organization noted between the cells. There were no visible morphological distinctions between the PBM groups and the control. The standard group, however, continued to show fibroblast-like morphology with evidence of nutrient depletion, as cells did not appear very healthy. Although diminished cell numbers were observed throughout the PBM groups compared to the mid time-point, 5 J/cm^2^ groups showed less thinning than the higher fluencies, with the combination 5 J/cm^2^ showing the least cell density amongst the other 5 J/cm^2^ fluence groups. Moreover, amongst all PBM groups, the combination 10 J/cm^2^ showed the least cell density, although spindle-like morphology was maintained.

### 3.2. Proliferation and Viability: ATP

**Figure 3:**
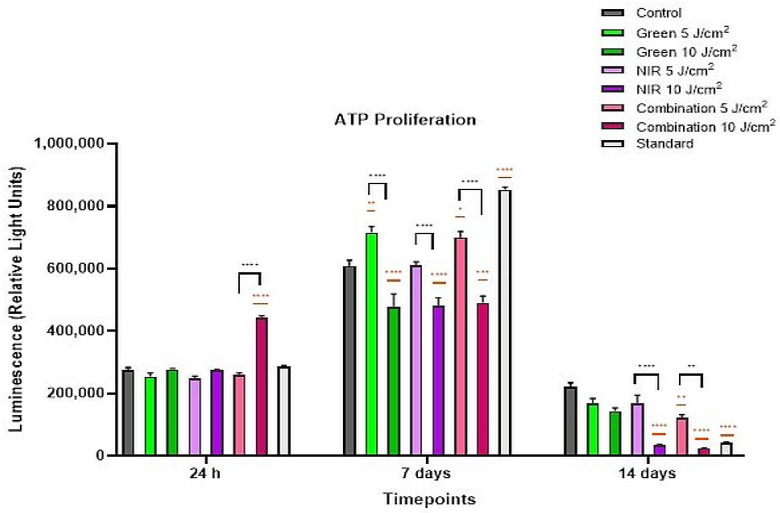
ATP luminescence assay of ADSCs exposed to PBM at 525 nm (G), 825 nm (NIR) and dual wavelengths (at 5 and 10 J/cm^2^ fluences) across 24 h, 7 days and 14 days. ATP levels, proportional to cellular proliferation and metabolic activity, were quantified via luciferase-based luminescence. At 24 h, the combination 10 J/cm^2^ showed significantly elevated ATP. By day 7, the standard group peaked, while 5 J/cm^2^ PBM groups maintained or increased ATP; all 10 J/cm^2^ groups showed significant reductions versus controls and their 5 J/cm^2^ counterparts. By day 14, ATP declined in all groups, with the greatest decrease observed in NIR and combination 10 J/cm^2^, and in the standard group. Statistical significance indicated as *p*< 0.05 to *p*< 0.0001. Black symbols indicate significance relative to the control group, while orange symbols indicate significance between fluences of the same wavelength.

The ATP luminescence assay was evaluated across the different groups and the fluences to evaluate the influence of PBM on the proliferation of the ADSCs and the reciprocal relationship with their metabolic activity across three time points. This assay uses the luciferase enzyme to generate a luminescent signal proportional to the concentration of ATP available in the sample. Cellular proliferation increases linearly with the amount of ATP in the sample, suggesting that increased levels of ATP are indicative of increased levels of both proliferation and, in turn, mitochondrial stimulation [30]. At 24 h, Figure 3 shows that most PBM groups displayed ATP levels comparable to the control, except for the combination 10 J/cm^2^ group, which showed a markedly elevated and statistically significant increase (*p* < 0.0001). A significant difference (*p* < 0.0001) was also observed between the combination 5 J/cm^2^ and 10 J/cm^2^ groups, indicating a fluence-dependent rise in ATP. At the intermediate time point, the standard exhibited the highest ATP levels relative to the control (*p* < 0.0001). Among the 5 J/cm^2^ PBM groups, all 5 J/cm^2^ treatments maintained or increased (G 5 J/cm^2^, *p* < 0.01 and combination 5 J/cm^2^, *p* < 0.05) ATP levels and thus proliferation; while, 10 J/cm^2^ fluences collectively showed reduced (G and NIR 10 J/cm^2^, *p* < 0.0001; and combination 10 J/cm^2^, *p* < 0.001) proliferative rates compared to both the control and their respective 5 J/cm^2^ fluences. Finally, by day 14, proliferation declined across all groups, including the control. Most 5 J/cm^2^ groups were slightly reduced, with the combination 5 J/cm^2^ showing the only significant decrease (*p* < 0.01). Interestingly, 10 J/cm^2^ groups, though also decreased, demonstrated markedly lower ATP levels than the control, particularly NIR and combination (both *p* < 0.0001), mirroring the low ATP levels also seen in the standard group. When comparing the different fluencies of the same wavelength groups, results reveal that the 10 J/cm^2^ fluencies of the NIR and combination groups showed a significant decrease (*p* < 0.0001 and *p* < 0.01, respectively) compared to their 5 J/cm^2^ groups.

### 3.3. Cell Cytotoxicity: LDH Assay

**Figure 4:**
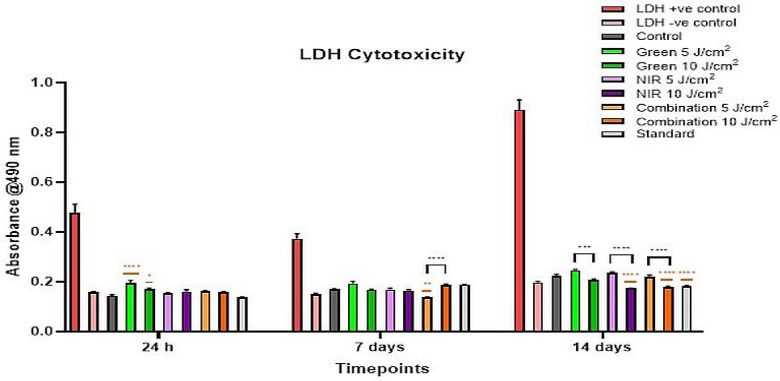
LDH cytotoxicity of ADSCs following PBM at 525 nm, 825 nm and a combination of both (at 5 and 10 J/cm^2^) over 24 h, 7 days and 14 days. LDH levels remained low relative to the positive control at all time points. G 5 and 10 J/cm^2^ showed slight increases at 24 h. By day 7, cytotoxicity was minimal, with combination 5 J/cm^2^ significantly reduced. At day 14, all 10 J/cm^2^ PBM groups showed significantly lower LDH than the control and their 5 J/cm^2^ counterparts, particularly NIR and combination (*p* < 0.0001). Black symbols indicate significance relative to the control group, while orange symbols indicate significance between fluences of the same wavelength.

LDH cytotoxicity was evaluated to determine PBM-related membrane damage across three timepoints. On initial assessment (Figure 4), all groups showed low LDH release far below the positive control. Only G 5 J/cm^2^ (*p* < 0.0001) and G 10 J/cm^2^ (*p* < 0.05) showed slightly elevated LDH, with no fluence-dependent differences within wavelengths. At day 7, cytotoxicity remained low across all groups. LDH levels were mostly similar to the control, with the G 5 J/cm^2^ showing a small, non-significant increase, and combination 5 J/cm^2^ showing a slight but significant decrease (*p* < 0.01). This group also had significantly lower LDH than its 10 J/cm^2^ counterpart (*p* < 0.0001). The standard group remained comparable to the control. At day 14, the positive control increased sharply while all other groups remained low. Clear fluence-related differences were observed with each of the 10 J/cm^2^ fluences showing reduced cytotoxic levels (G 10 J/cm^2^, *p* < 0.001; NIR and combination, *p* < 0.0001) than their respective 5 J/cm^2^ fluence groups. The standard LDH cytotoxicity levels are also significantly decreased (*p* < 0.0001) compared to the control at this final time point.

### 3.4. Mitochondrial Membrane Potential

**Figure 5:**
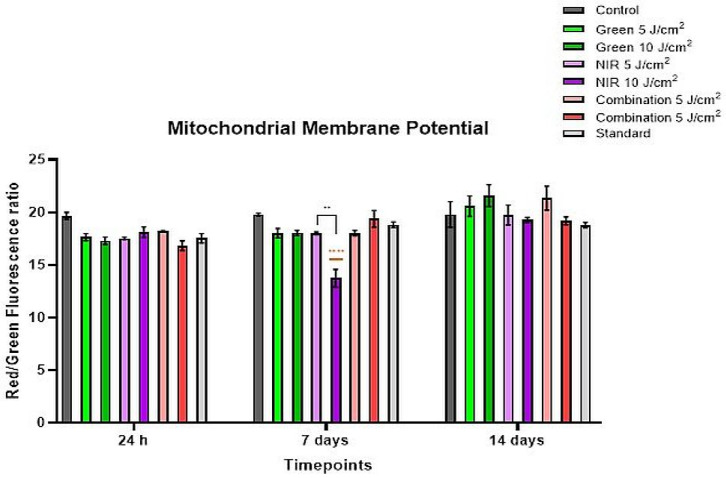
Mitochondrial Membrane potential (MMP) Ratios of ADSCs exposed to PBM across three timepoints. At 24 h, the control group displayed the highest MMP ratio, with all PBM and standard groups showing similarly lower but non-significant values. Combination 10 J/cm^2^ showed a slight decrease yet remained comparable to other PBM groups. At day 7, green (5 J/cm^2^ and 10 J/cm^2^) and NIR 5 J/cm^2^ trended lower than the control, while NIR 10 J/cm^2^ demonstrated a significant reduction versus both the control (*p* < 0.0001) and its 5 J/cm^2^ fluence (*p* < 0.01). Combination 10 J/cm^2^ remained close to control levels. By day 14, most PBM groups exhibited higher MMP ratios than both control and standard, although without statistical significance. Across all timepoints, the standard consistently remained below the control. Black symbols indicate significance relative to the control group, while orange symbols indicate significance between fluences of the same wavelength.

Initially, Figure 5 shows that the control group MMP ratio is higher than all other groups, including the standard, without differing significantly, and thus, there is no detectable PBM-related MMP effect at an early onset. The combination 10 J/cm^2^ group in particular shows a slightly decreased ratio, yet insignificant compared to other PBM groups. At the subsequent assessment, greenlight at both fluences and NIR 5 J/cm^2^ trend lower than the control, in the absence of statistical significance. NIR 10 J/cm^2^; however, it particularly stands out since not only is it significantly reduced (*p* < 0.0001) compared to the control, but it is also significantly lower than its 5 J/cm^2^ counterpart (*p* < 0.01). Unlike other 10 J/cm^2^ fluence, the combination 10 J/cm^2^ is closer in range to the MMP ratio of the control or only slightly lower. It is also visibly higher than its 5 J/cm^2^ counterpart, albeit without significance. At this stage, the standard group remains lower than the control group and only slightly higher than its initial MMP ratio at day 1. Finally, at the latest stage, most PBM groups reflect MMP ratios higher than both the control group and the standard, although the differences are not flagged as significant, the increasing trend is noticeable. Lastly, over the three timepoints, the standard consistently remained lower than that of the control group.

### 3.5. Collagen

**Figure 6:**
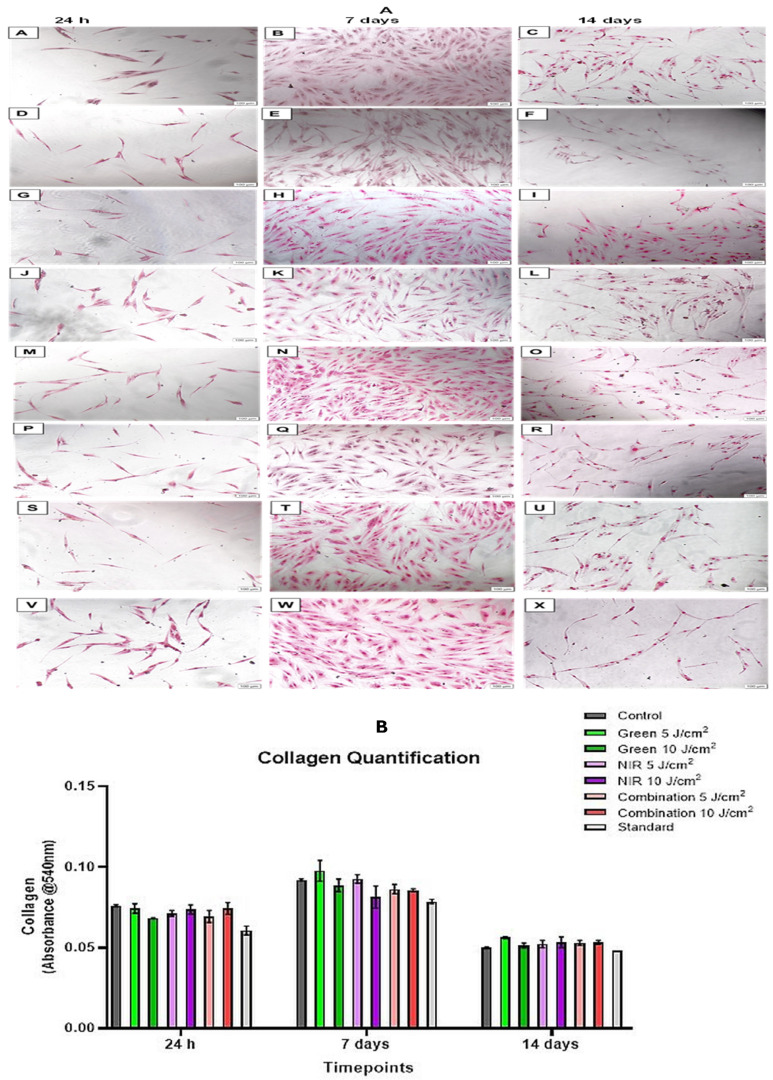
Collagen deposition in ADSCs following PBM treatment. (**A**) Qualitative Sirius red staining of collagen deposition in ADSCs following PBM (10X magnification). Here, Sirius red-stained images show collagen distribution across treatment groups: Control (**A**–**C**), standard (**D**–**F**), G 5 J/cm^2^ (**G**–**I**), G 10 J/cm^2^ (**J**–**L**), NIR 5 J/cm^2^ (**M**–**O**), NIR 10 J/cm^2^ (**P**–**R**), combination 5 J/cm^2^ (**S**–**U**) and combination 10 J/cm^2^ (**V**–**X**). Initially, collagen staining intensity appears comparable across groups, with slightly reduced deposition in G 10 J/cm^2^ and combination 5 J/cm^2^, consistent with quantitative results. By day 7, all PBM groups show increased collagen accumulation, most pronounced in G 5 J/cm^2^, while the standard remains lowest. At day 14, collagen staining decreases overall but remains mildly higher in PBM groups relative to control, particularly in G 5 J/cm^2^. The standard consistently exhibits the weakest staining across all timepoints. All images are at a 10× magnification with a scale bar of 100 µm. (**B**) Collagen quantification of ADSCS following PBM at 525 nm, 825 nm and dual wavelengths across three timepoints. At 24 h, collagen levels in most PBM groups were comparable to the control, with slight decreases in G 10 J/cm^2^ and combination 5 J/cm^2^, and the lowest levels in the standard. By day 7, collagen increased across all groups, with G 5 J/cm^2^ exceeding the control and the lower fluences (G 5 J/cm^2^, NIR 5 J/cm^2^) trending higher than 10 J/cm^2^. Combination fluences remained slightly below the control. By day 14, collagen declined across all groups, though all PBM treatments remained slightly higher than control, with G 5 J/cm^2^ highest. The standard remained lowest throughout.

Qualitatively, Figure 6A shows that collagen staining was initially similar across groups but slightly lower in G 10 J/cm^2^ and combination 5 J/cm^2^. It increased by day 7, particularly G 5 J/cm^2^, then decreased by day 14 while remaining slightly higher in PBM groups, with the standard consistently showing the weakest staining. Quantitative collagen analysis, however, (Figure 6B), the first 24 h, collagen levels of most PBM groups were relatively similar to the control, with G 10 J/cm^2^ and combination 5 J/cm^2^ being slightly but non-significantly lower. The standard groups expressed the lowest collagen levels at the initial time point, although not statistically significant compared to the control. Moreover, in the case of NIR and combination light, the 10 J/cm^2^ fluences showed slightly increased collagen levels than the lower fluences, though not significantly. By day 7, collagen levels increased across all experimental groups, with the levels G 5 J/cm^2^ surpassing that of control levels and the standard remaining the lowest of all the experimental groups. Notably, this time around, the lower fluences (G and NIR) expressed increased collagen levels compared to their 10 J/cm^2^ groups. In the case of the combination, both fluences expressed similar collagen levels to one another, although insignificantly lower than the control. Finally, at day 14, collagen levels subsided across all experimental groups, with all PBM levels slightly higher than the control, the G 5 J/cm^2^ being the highest of them all, albeit without significance. The standard remained the group with the lowest collagen levels expressed in comparison to the control across all three timepoints. Both fluences across the groups were relative to one another, except in the case of greenlight, where the 5 J/cm^2^ fluence was higher than the 10 J/cm^2^ in the absence of significance.

### 3.6. Cell Migration

**Figure 7:**
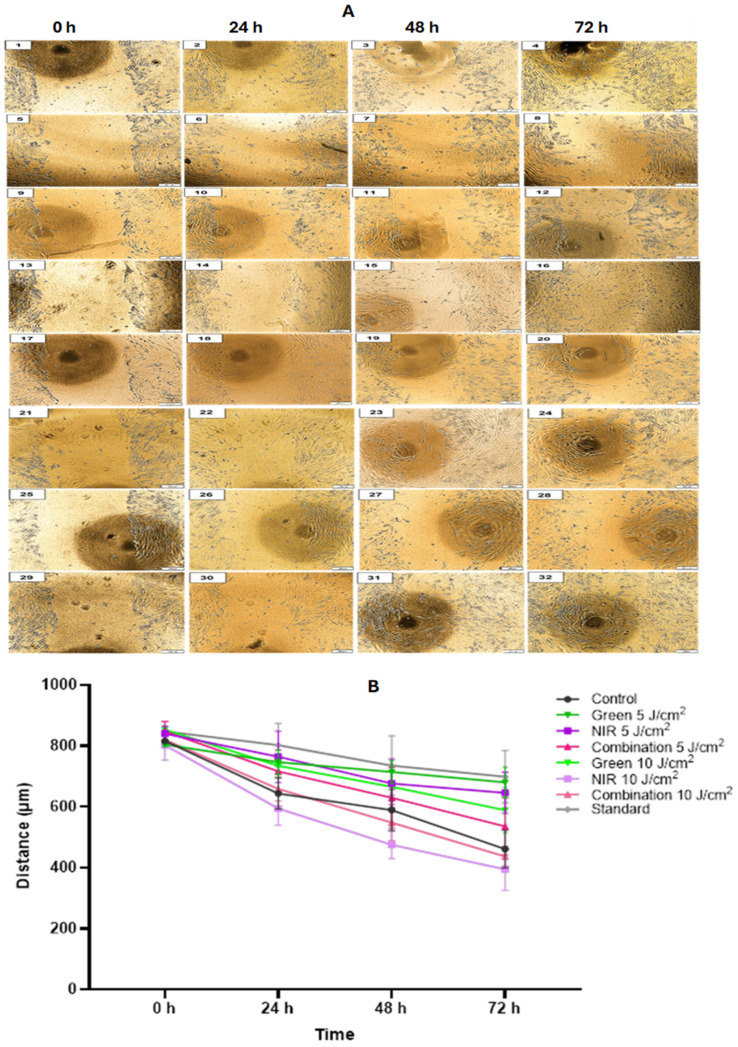
Scratch wound migration assay of ADSCs following PBM treatment. (**A**) Qualitative central scratch assay images of ADSCs migration following PBM (4× Magnification with 200 µm scale bar). Representative microscopic images show wound closure overt 0, 24, 48 and 72 h across all treatment groups: Control (**1**–**4**), standard (**5**–8), G 5 J/cm^2^ (**9**–**12**), G 10 J/cm^2^ (**13**–1**6**), NIR 5 J/cm^2^ (**17**–**20**), NIR 10 J/cm^2^ (**21**–**24**), combination 5 J/cm^2^ (**25**–**28**), and combination 10 J/cm^2^ (**29**–**32**). Images demonstrate the progressive reduction in scratch width consistent with quantitative migration trends. The control shows steady closure, while the standard demonstrates the slowest wound reduction. NIR 10 J/cm^2^ displays the most pronounced closure over time, followed by combination 10 J/cm^2^. G 5 J/cm^2^, G 10 J/cm^2^, NIR 5 J/cm^2^ and combination 5 J/cm^2^ exhibit varying but generally slower closure relative to their higher-fluence groups. (**B**) Quantitative analysis of scratch-wound migration of ADSCs at day 14 following PBM over 72 h. Wound distance (µm) was measured at 0, 24, 48 and 72 h, where smaller distances indicate faster migration. All groups began with similar baseline widths. At 24 h, NIR 10 J/cm^2^ showed the greatest early migration, while the standard and G 5 J/cm^2^ remained slowest. At 48 h, NIR 10 J/cm^2^ significantly outperformed NIR 5 J/cm^2^ (*p* < 0.05) and all other groups. At 72 h, the standard remained significantly slower than control (*p* < 0.01), and G 5 J/cm^2^ showed reduced migration (*p* < 0.01). NIR 10 J/cm^2^ exhibited the fastest overall migration (*p* < 0.001 vs. NIR 5 J/cm^2^). Combination 10 J/cm^2^ exceeded control, while 5 J/cm^2^ remained slower. In the above assay, *n* = 3.

A central scratch assay was used to evaluate ADSCs migration qualitatively (Figure 7A) and quantitatively (Figure 7B), with the latter at the day-14 timepoint over 72 h, where smaller remaining wound distances indicate faster migration. At the 0 h, all groups began with comparable scratch widths. By 24 h, early trends emerged without statistical significance. The control showed moderate closure reflecting strong motility with growth factors. The standard migrated slowly, likely due to low serum and lack of cues. G 5 J/cm^2^ and 10 J/cm^2^ showed similar distances, both slightly slower than the control. NIR 5 J/cm^2^ migrated less than the control, while NIR 10 J/cm^2^ displayed the largest early closure. Combination 10 J/cm^2^ approximated control performance, whereas combination 5 J/cm^2^ lagged behind. At 48 h, the standard remained slowest. G 5 J/cm^2^ migrated less than G 10 J/cm^2^, though both remained behind the control. NIR 10 J/cm^2^ showed significantly greater migration than NIR 5 J/cm^2^ (*p* < 0.05) and was the fastest of all groups. Combination 10 J/cm^2^ ranked second fastest, while combination 5 J/cm^2^ remained slower than control. By 72 h, the control was the third fastest group. The standard remained significantly slower (*p* < 0.01). G 5 J/cm^2^ showed significantly reduced migration relative to control (*p* < 0.01), while G 10 J/cm^2^ improved but remained below control. NIR 10 J/cm^2^ again showed the greatest overall migration (*p* < 0.001 vs. NIR 5 J/cm^2^). Combination 10 J/cm^2^ exceeded control, whereas combination 5 J/cm^2^ trailed behind. Qualitative results in Figure 7A below reflected the quantitative analysis. Table 2 reflects the scratch assay performed at day 14 over 72 h. Significances indicated with a * elutes to the significance compared to the control group, while # indicates the significance between fluences of the same wavelength.

### 3.7. ADSCs Characterization and SMC Markers

**Figure 8:**
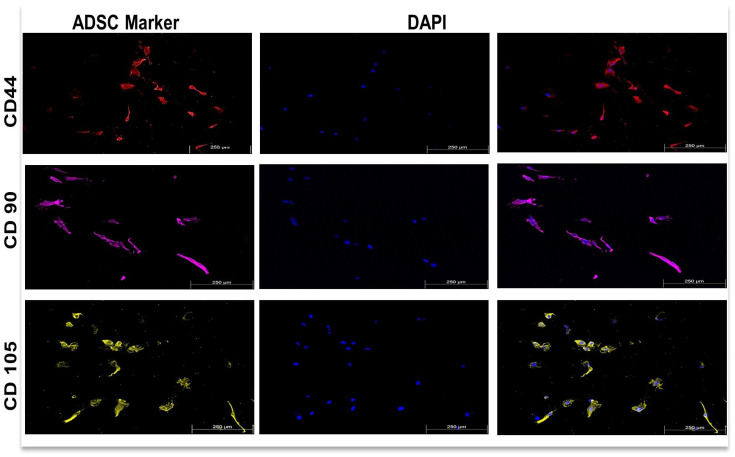
Immunofluorescent characterization of ADSCs. After three days of culture in complete medium (10% FBS, no growth factors), ADSCs exhibited positive membrane-associated fluorescence for stemness due to the presence of characteristic mesenchymal surface markers CD 44, CD 90 and CD 105, confirming their mesenchymal identity. All images are at a 10× magnification with a scale bar of 250 µm.

ADSCs were characterized for stemness using the following characteristic surface markers through immunofluorescent techniques: CD44, CD90 and CD105. Figure 8 below shows that following three days of culture in a plastic T75 flask containing complete media with 10% FBS and void of any induction and PBM, ADSCs showed positivity for all three cell surface markers, confirming their mesenchymal identity.

**Figure 9:**
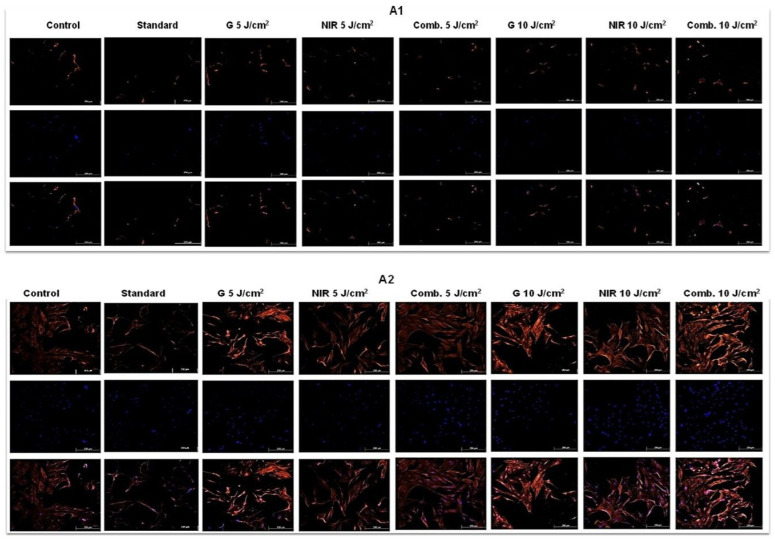

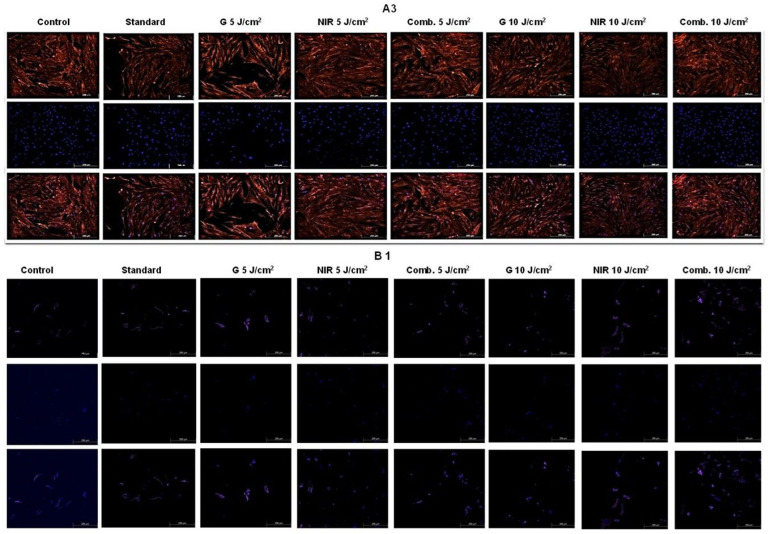

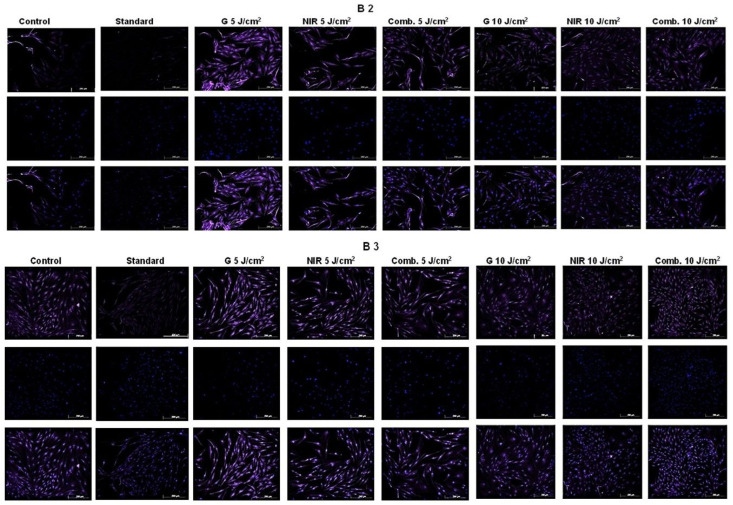

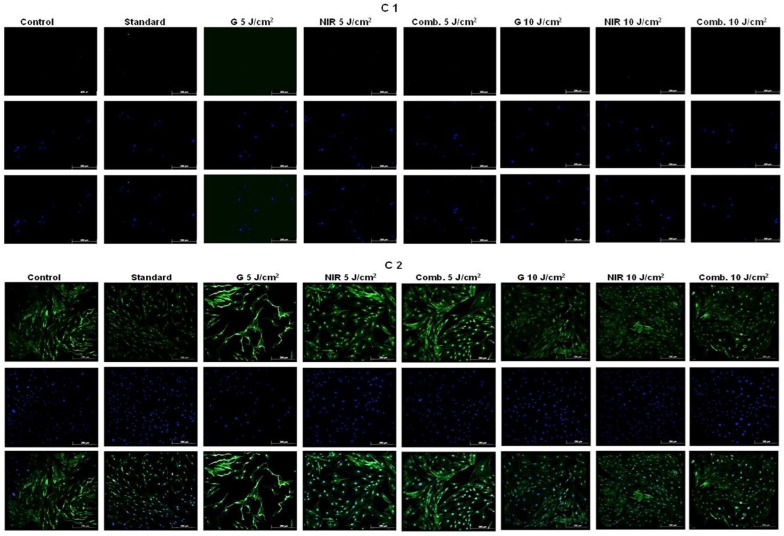

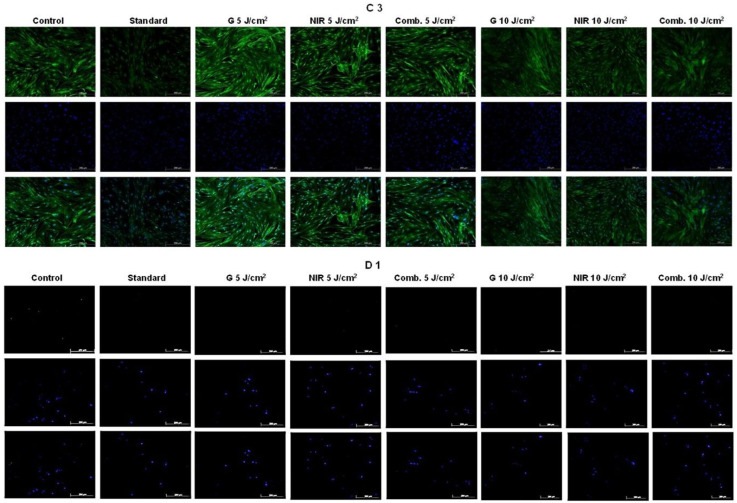

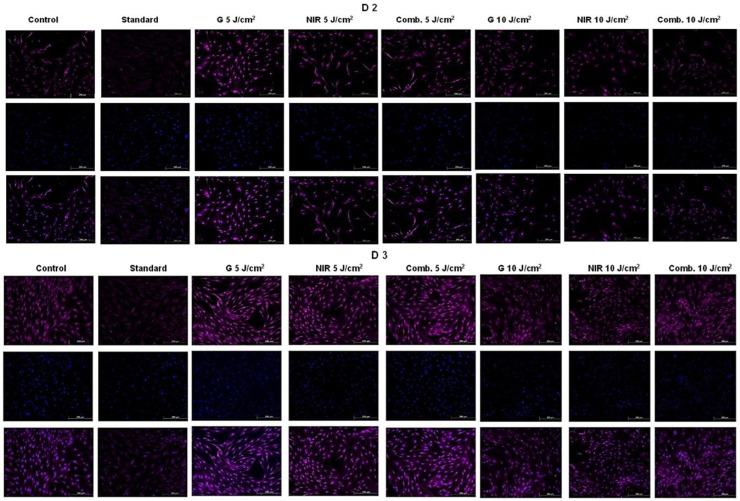
Immunofluorescence staining of SM markers SMAα (**A1**–**A3**), Desmin (**B1**–**B3**), Calponin (**C1**–**C3**) and SMMHC (**D1**–**D3**) at 24 h, 7 days and 14 days. At 24 h, all groups showed minimal staining, indicating early induction with limited cytoskeletal organization. By 7 days, the control and all 5 J/cm^2^ PBM groups display stronger filamentous SMAα, increased desmin and clear calponin fibres, while SMMHC becomes faintly detectable, marking the onset of terminal differentiation. Contrastingly, 10 J/cm^2^ groups exhibit weaker, less organized staining across markers at this stage. By 14 days, SMAα, desmin and calponin appear intensely filamentous in the control, 5 J/cm^2^ groups, accompanied by strong SMMHC expression, consistent with a more advanced contractile-like state in SMC-like cells. The standard and 10 J/cm^2^ groups show comparatively weaker organization and reduced SMMHC intensity. G 5 J/cm^2^ consistently showed appropriate levels and intensity of SMC markers throughout the developmental stages. The observed nuclear or perinuclear signal localisation in some images is attributed to the close organization of cytoskeletal proteins around the nucleus and partial overlap with DAPI counterstaining, rather than true nuclear expression—scale: 250 µm with 10× magnification.

With regard to SMC markers, across all markers, a distinct pattern emerged reflecting both time-dependent maturation and fluence-dependent PBM effects as demonstrated in Figure 9. At 24 h (A1–D1), all groups showed minimal SMAα, desmin, calponin and SMMHC expression; with SMAα occurring in all experimental groups, albeit faint and scattered in the standard and 10 J/cm^2^ groups, while more intense in the 5 J/cm^2^ groups. By day 7, (A2–D2) marker upregulation became more group-specific. The 5 J/cm^2^ groups, particularly G 5 J/cm^2,^ exhibited strong filamentous SMAα and moderate desmin organization, indicating early cytoskeletal assembly. These same 5 J/cm^2^ groups also showed the clearest calponin fibres, while SMMHC became detectable but still low, reflecting early terminal commitment. In contrast, the G 10 J/cm^2^, NIR 10 J/cm^2^ and combination 10 J/cm^2^ groups displayed weaker calponin and SMMHC staining, suggesting delayed or stress-affected differentiation. By day 14 (A3–D3), the control, G 5 J/cm^2^, NIR 5 J/cm^2^ and combination 5 J/cm^2^ groups exhibited intense, fully organized networks of SMAα, desmin and calponin, accompanied by strong, widespread SMMHC, indicating a contractile-like SMC phenotype. The standard group remained minimally positive across all markers, while all 10 J/cm^2^ groups showed increased marker expression relative to day 7 but still noticeably weaker and less organized than the 5 J/cm^2^ groups, consistent with a partially differentiated or stress-modulated phenotype. A summary of key findings for each assay has been represented in Table 3 below.

## 4. Discussion

When referring to PBM, we induce the understanding of low-level light at various wavelengths, of which its irradiation may be either continuous or pulsed, with the supply of low and constant energy density aimed at a specific tissue or cell monolayer, with the use of powers measured in mW [31]. This study looked into how PBM at 525 nm (G), 825 nm (NIR) and dual wavelength combination influences ADSC proliferation, viability, mitochondrial function, extracellular matrix production, migration and differentiation into SMC in the presence of growth factors such as RA and TGF-β. This was accomplished by performing metabolic assays, cytotoxic evaluations, quantitative and qualitative collagen analysis, migration analysis and assessing immunofluorescence of ADSC stemness and SMC markers, allowing for a clear picture as to how PBM modulates ADSCs in a fluence-dependent manner.

Morphological analysis showed that while all groups initially exhibited typical spindle-shaped ADSCs, fluence-dependent effects became evident at later timepoints. Lower fluence (5 J/cm^2^) maintained higher cell density and healthy morphology, whereas 10 J/cm^2^ groups displayed reduced proliferation and sparser cultures, particularly in the combination group. These observations are consistent with Arndt-Schulz’s law, which states that a weak stimulus is capable of slightly accelerating vital cellular activity while moderate stimuli can augment activity; however, at a given moment, a peak is reached, and thereafter, if stimuli increase, a suppression will occur until an inhibitory response occurs. The results align with the classical biphasic response, where lower fluences stimulate proliferation while higher fluences suppress growth or induce metabolic stress. The results obtained in the present study align with the aforementioned law. Moreover, the standard group consistently showed fibroblast-like morphology with thin cellular extensions and signs of nutrient depletion, the latter being a feat observed in another study conducted by Ciaverella et al. (2015) when mesenchymal stem cells derived from abdominal aortic aneurysms were subjected to complete nutrient depletion and exhibited contracted cellular volume with loss of typical cellular shape [32]. This highlights the importance of growth factors required for maintaining ADSC health and promoting differentiation.

ATP results supported this biphasic response, with early increases in high-fluence combination groups likely reflecting transient metabolic stress rather than true proliferative enhancement. At day 7, the biphasic response became clearer with all the lower fluence groups showing equal or higher ATP levels compared to the control with G 5 J/cm^2^ (*p* < 0.01) and combination 5 J/cm^2^ (*p* < 0.050) being the most significantly elevated finding that align with those of Crous et al. (2022) where G and combination 5 J/cm^2^ increased after 7 days of measurement in 5 J/cm^2^ fluences [30]. In contrast, the 10 J/cm^2^ groups showed reduced ATP levels compared to both the control and their 5 J/cm^2^ counterparts (*p* < 0.0001). Finally, at the last time point, a decrease in proliferation in all groups was observed, suggestive of two possible cases. The first is nutrient depletion since cells have overcrowded in a tight space from day 7 to this time, resulting in limited space and nutrients in the culture plate [33]. Secondly, a decrease in proliferative levels, particularly in the PBM groups and the control group, points to differentiation-associated metabolic slowing arising from the redirection of cellular energy from proliferation to differentiation. Our observations are consistent with several studies, including that of Mvula et al. (2014) and Da Silva and colleagues (2023). Both authors observed a decline in proliferation of ADSCs with the initiation of differentiation of SMC and osteogenic cells in their respective studies, specifically at lower PBM doses [23,33]. Similar biphasic PBM effects have been reported in fibroblasts, where low doses stimulate cell proliferation and higher doses inhibit cellular activity [31] highlighting that lower fluence supports metabolic activity, where higher fluence suppresses it. Interestingly, these studies utilized both primary and immortalized ADSCs, yet demonstrated similar trends in PBM-induced differentiation, suggesting that the observed effects are reproducible across different ADSC models.

Across all timepoints, LDH levels remained low relative to the positive control, confirming that PBM at either fluence did not produce any cytotoxicity. Notably, at 24 h, only G 5 J/cm^2^ (*p* < 0.0001) and G 10 J/cm^2^ (*p* < 0.05) mildly elevated LDH, whereas at day 7, the combination 5 J/cm^2^ exhibited significantly *lower* LDH levels compared to both the control (*p* < 0.01) and the combination 10 J/cm^2^ (*p* < 0.0001), an indication of greater membrane stability at lower fluence. Moreover, by day 14, higher fluence groups, particularly NIR and combination, showed lower LDH than the control (*p* < 0.0001). This late-stage LDH decline in 10 J/cm^2^ groups may reflect reduced cell turnover and motility rather than true “improved viability” coinciding with the lower ATP results and sparser morphology seen at the same time point.

Mitochondrial membrane potential arises from proton movement during oxidative phosphorylation and is crucial for sustaining the electrochemical gradient necessary for ATP generation. Maintaining a steady MMP is vital for normal cell activity, with any changes generally signalling shifts in cellular metabolism or physiological state. In PBM, irradiation can modulate the electron transport chain by dissociating nitric oxide from cytochrome C oxidase, therefore enhancing mitochondrial polarization [33]. Increased MMP is strongly associated with greater differential potential. The red-to-green fluorescence ratio of the dye in mitochondria reflects the degree of mitochondrial polarization. A higher mitochondrial membrane potential produces a stronger red shift due to increased formation of J-aggregates, whereas a lower potential results in fewer aggregates and a reduced red-to-green fluorescence ratio altogether. Consequently, mitochondrial depolarization is indicated by a decrease in the red-to-green fluorescence intensity ratio [34]. Several studies demonstrate that elevated MMP is a necessary event in mesenchymal stem cell differentiation as cells adapt to the increased energetic demand [33]. Da Silva et al. (2023) reported sustained MMP in ADSCs cultured in osteogenic induction medium over 48 h and 7 days, with a significant increase in the NIR-G 5 J/cm^2^ group [33]. In the same study, cells maintained in induction media showed sustained MMP over time in the control group, indicating the positive effect of induction media in lineage commitment. Crous and colleagues (2022) similarly observed enhanced MMP following NIR-G exposure at a similar wavelength and time point [30].

In contrast, during the first 7 days of the present study, MMP ratios across PBM groups portrayed a low, non-significant trend in comparison to the control, suggestive of a minimal early mitochondrial response. By day 7, only the NIR 10 J/cm^2^ group showed a significant MMP decline, consistent with mitochondrial depolarization and corresponding reduced ATP levels. This significant decrease can be attributed to high PBM doses (>10 J/cm^2^) having an inhibitory effect, as supported by Pchelin and colleagues (2022) [35]. Moreover, the causative mechanism of this inhibitory effect is a result of high NIR dose capable of destroying cytochromes and ultimately inhibition resulting from excessive production of reactive oxygen species [28]. Although limited literature exists at comparable fluences, Zein et al. (2018) noted that ineffective PBM outcomes are more often due to overdosing than underdosing, and that excessive light exposure can reduce MMP below baseline levels after surpassing an optimal threshold [36]. By day 14, most PBM groups exhibited a higher, non-significant trend of MMP ratios than the control, despite the natural ATP decline, suggesting improved mitochondrial polarization during later differentiation. Green light at both fluences and NIR-G at 5 J/cm^2^ produce the highest MMP values, the latter consistent with Da Silva and Crous. Our findings indicate that PBM maintains or enhances MMP during later maturation, with green light demonstrating sustained benefits and NIR-G at 5 J/cm^2^ supporting improved mitochondrial function as cells approach a more contractile-like SMC resembling phenotype. In a review by Pearce (2024), the author mentions the reversible transition between contractile SMC and the noncontractile phenotypes being influenced by various factors, including the mitochondria, further indicating that mitochondrial proteins promote contractile differentiation [37] hence suggesting that healthy mitochondria and thus potential are necessary for contractile SMC phenotypes.

SMCs display phenotypic plasticity and are unique within the myogenic family since they maintain multiple functional abilities, including contraction, proliferation, migration, growth factor secretion and ECM production [38]. The ECM can be defined as a noncellular component that surrounds vascular as well as other organs. SMCs, in particular, are surrounded by a basement membrane consisting of ECM proteins, including laminins, collagen, perlecans and other ECM proteins and are embedded in fibrillar collagen types I, III and V [39]. In contrast to striated muscles, SMCs are able to shift from a proliferative, “synthetic” state to a highly contractile state. This bidirectional transition–known as phenotypic modulation–is a well-recognized feature of mature SMC across all visceral and vascular tissues studied [38]. Modulation of SMC from a contractile to a synthetic phenotype is directly associated with an increase in protein synthesis, particularly collagen. Type I collagen is predominantly found in all SMC phenotypes, with the synthetic-state SMC producing more type I collagen (91%) than the contractile state (78%) [40]. SMC can shift reversibly along the notion of a quiescent, contractile phenotype to a synthetic phenotype characterized by proliferation and ECM synthesis [41]. This aligns with our findings in this study, initial results saw collagen levels being comparable to the control at 24 h with an average non-significant increase; however, the G 5 J/cm^2^ group was the highest amongst all the groups, although statistically without significance. The 10 J/cm^2^ PBM groups were also visually lower than the control, and in comparison, to their 5 J/cm^2^ counterparts; however, this was not statistically significant. Later, collagen levels of all groups declined, with the G 5 J/cm^2^ group still greater than the control, although without significance, indicating that at the midpoint, cells may have been undergoing ECM remodelling with upregulation of ECM proteins such as collagen and thus may have been in a synthetic-like state and later at 14 days switched to a contractile-like phenotype where the reverse is true.

A phenotypic modulation from contractile to synthetic has been made a prerequisite for vascular SMC migration. Synthetic SMCs portray increased capabilities for migration, proliferation and protein synthesis, of which these two phenotypes convert reversibly in culture conditions [42]. In vitro, the loss of SMC contractile phenotype (to a synthetic one) is reflected through reduced levels of SMC marker genes as well as increased levels of cellular migration and proliferation. In normal healthy vessels, it has been observed that SMCs are found in a differentiated or contractile state where they are quiescent with no migration; however, increased migratory and proliferative levels are signs of SMC phenotypic switching, along with decreased SMC contractile marker genes. This is supported by several studies, in one study where phenotypic responses of vascular SMC where assessed when exposed to mechanical cues, phenotypic switching was noted and accompanied by proliferation, and migration of SMC after shear stress [39], in another study where the effect of palmitic acid on vascular SMC synthetic phenotype was researched, contractile phenotype was confirmed due to decreased proliferation and migration [43]. In this experiment, migration results (Figure 7) further confirmed functional differences among the groups, with strong differences in motility between fluences. The standard showed the slowest migration at all timepoints (*p* < 0.01 at 72 h), confirming poor motility in the absence of GF and coinciding with lower ATP, MMP and collagen. G 5 J/cm^2^ displayed reduced migration at 48 and 72 h compared to the control, with the latter at 72 h being significant (*p* < 0.01), consistent with its strong ECM deposition and organized cytoskeletal maturation–traits typical of a contractile-like state of SMC-like, which migrate slowly. NIR 10 J/cm^2^ consistently demonstrated the highest migration at 48 and 72 h and significantly exceeded NIR 5 J/cm^2^ (*p* < 0.05), indicating a shift towards a more motile, synthetic-like phenotype under higher fluence. In both green and NIR groups, 10 J/cm^2^ promoted greater migration than the 5 J/cm^2^ groups. Thus, 5 J/cm^2^ groups potentially acquired a more contractile-like phenotype, while the 10 J/cm^2^ groups, particularly NIR 10 J/cm^2,^ behaved more like synthetic-like SM-like cells, consistent with ATP/MMP suppression and earlier cytoskeletal response. Normalization of the scratch assay, demonstrating the remaining scratch over the 72 h, has been depicted in Table 2 to further highlight motility differences. The increased migration observed at higher fluence due to synthetic-like phenotypic characteristics can be attributed to cells exhibiting marked phenotypic plasticity in response to altered environmental cues, with the change to a migratory phenotype occurring under stress or changing conditions [44,45]. A likely contributing factor is reactive oxygen species generated during PBM, which act as signalling molecules that regulate vascular SMC-like behaviour including migration [46,47]. The increased reactive oxygen species signalling has been linked with cytoskeletal reorganization as well as the activation of promigratory signalling pathways in vascular SMC and thus has been implicated in phenotypic modulation, particularly that of a more synthetic, migratory state [48]. Altogether, it is likely that higher PBM dose enhanced redox signalling sufficiently to favour pathways associated with migration rather than contractile-like maturation.

Although cellular metabolism is implicated in SMC phenotypic modulation, there remains a lack of direct evidence linking ATP alone to contractile-synthetic switching, with current literature instead emphasizing mitochondrial function and metabolic reprogramming as key regulators of phenotype. Mitochondrial dysfunction has been associated with SMC phenotypic alteration, while the synthetic phenotype is characterized by increased migration and ECM remodelling [49,50]. In our study, a fluence- and time-dependent response was observed. Lower fluence (5 J/cm^2^) maintained higher ATP at early and intermediate timepoints, preserved MMP, and promoted increased collagen production, particularly at day 7, supporting a metabolically active, ECM-producing state consistent with a more contractile-like phenotype. In contrast, higher fluence (10 J/cm^2^) showed reduced ATP and collagen, but increased migration at day 14, indicative of a more synthetic-like phenotype. Since migration was only assessed at day 14, its temporal relationship with ATP cannot be directly established. However, when interpreted alongside ATP, MMP, and collagen trends, the findings suggest that migration is not directly proportional to ATP levels but rather reflects phenotypic differences between groups. Therefore, ATP should be interpreted as a marker of the cellular metabolic state supporting phenotype-specific functions such as ECM production, rather than a direct determinant of migration or SMC phenotypic switching.

ADSCs comprise a subset of MSCs with multipotent abilities; they have been extensively studied in recent years due to their attractiveness in the ease of obtaining these cells in large numbers, along with their high regenerative potential and immunomodulatory characteristics. In vitro, mesenchymal identity is confirmed with their ability to adhere to plastic cell culture flasks under standard culture conditions as well as their expression of surface antigens, including but not limited to CD 44, CD 90 and CD 105, whilst lacking haematopoietic lineage markers (CD 13, CD 19, CD 45). These stem cells can differentiate into various cell lines, including adipocytes, osteocytes and myogenic lineages under suitable in vitro culture conditions [38,51,52]. In this study, we were able to successfully characterize ADSCs as MSC phenotype consistent with the literature due to positive CD 44, CD 90 and CD 105 surface markers expression (Figure 8). hTERT-immortalized ADSCs were used to ensure experimental consistency and retain key stem cell properties, although immortalization may slightly alter certain cellular characteristics compared to primary ADSCs; these variations were not observed [53]. Following simultaneous induction with RA and TGF-β, with or without PBM, ADSCs progressed through distinct SM differentiation stages that corresponded closely with metabolic, ECM and functional outcomes.

SMAα and desmin represent early, although non-specific early myogenic or SMC differentiation markers, whilst calponin constitutes an intermediate SMC marker and SMMHC is a specific late SMC differentiation marker [38,54,55,56,57]. During the initial days, SMAα, desmin, calponin and SMMHC expression was minimal across all groups, indicating that early lineage priming was underway; however, structural remodelling was still limited. By day 7, the control and 5 J/cm^2^ groups exhibited prominent filamentous SMAα and increasing desmin organization, accompanied by emerging calponin and faint SMMHC signals corresponding to an intermediate or modulated SMC phenotype. Aligning with metabolic findings of moderate ATP activity, reduced yet stable MMP and increased collagen deposition typical of the synthetic-to-contractile transition. According to several studies, vascular SMCs possess inherent plasticity allowing them to adopt states with both proliferative and ECM-secreting behaviour where they reflect high abilities of proliferation, migration and ECM secretion, such as collagen and elastin; moreover, during phenotypic modulation, SMCs undergo downregulation of contractile proteins such as SMAα, calponin and SMMHC, supporting our observations [58,59]. Contrastingly, all 10 J/cm^2^ PBM groups exhibited weaker marker expression at day 7, consistent with reduced metabolic activity and delayed differentiation. By day 14, control and all 5 J/cm^2^ groups displayed strong, filamentous expression of all markers, particularly calponin and SMMHC, confirming a contractile-like phenotype. This was supported by reduced migration, stabilized MMP, and decreased ATP, likely reflecting a shift from proliferation to differentiation [60]. Conversely, NIR 10 J/cm^2^ and combination 10 J/cm^2^ groups maintained weaker marker expression and higher migration, indicating persistence of a synthetic-like phenotype, although G 10 J/cm^2^ showed partial recovery at later stages. While the present study demonstrates consistent trends across multiple independent parameters, including morphology, mitochondrial function, ECM production and reduced migration, these findings should be interpreted as indicative of SMC-like differentiation rather than definitive confirmation of a fully mature contractile phenotype. Quantitative gene or protein expression analyses and functional contractility assays would be required to conclusively validate terminal differentiation. This study has several limitations. Smooth muscle differentiation was assessed primarily using qualitative immunofluorescence and indirect functional indicators, without quantitative gene or protein validation. Functional contractility assays were not performed, limiting confirmation of a fully mature contractile phenotype. Additionally, the use of a two-dimensional culture system does not fully replicate the in vivo microenvironment, and immortalized ADSCs may not entirely reflect primary cell behaviour. Future studies incorporating three-dimensional models, quantitative analyses, and functional assays are required to further validate these findings.

## 5. Conclusions

This study demonstrates that PBM, when combined with RA and TGF-β, significantly influences ADSC behaviour and their progression towards a SMC-like phenotype in a fluence-dependent manner. Across metabolic, structural and functional assays, 5 J/cm^2^ fluences consistently supported enhanced proliferation, mitochondrial stability, ECM production, cytoskeletal organization and expression of contractile-like SM markers. Culminating in an SMC-like phenotype with features consistent with a more contractile-like state by day 14. In contrast, 10 J/cm^2^ fluences imposed metabolic strain, reflected by reduced ATP, decreased early MMP, diminished collagen production, delayed marker expression and increased migration, features consistent with a more synthetic-like or stress-modulated phenotype, potentially delaying contractile maturation. These findings highlight the importance of precise PBM dosing for directing ADSC fate and identify 5 J/cm^2^ PBM, particularly 525 nm greenlight, as a favourable parameter for promoting functional SMC-like differentiation, of which several mechanisms may potentially be responsible for this. While red and NIR wavelengths are traditionally associated with PBM through CCO, emerging evidence suggests that alternative mechanisms may explain the effectiveness of shorter wavelengths. Greenlight has been shown to enhance stem cell differentiation through mechanisms involving intracellular calcium signalling, where activation of light-gated ion channels increases calcium levels and promotes differentiation pathways [61]. Additionally, PBM-induced production of intracellular reactive oxygen species, ATP, and nitric oxide is fundamental in regulating differentiation processes [62]. Interestingly, greenlight has also been associated with modulation of intracellular reactive oxygen species levels, further supporting its role in signalling-mediated differentiation. Moreover, PBM effects are not limited to CCO alone, as other chromophores such as transient receptor potential channels, flavins, and opsins may contribute to cellular responses at shorter wavelengths [63]. Together, these mechanisms may account for the enhanced differentiation observed at 525 nm despite its lower interaction with classical mitochondrial photoacceptors. The combination group did not necessarily outperform single-wavelength irradiation, with G 5 J/cm^2^ yielding the most favourable outcomes, suggesting that optimal effects are achieved under specific wavelengths and dose conditions, and increased treatment complexity does not guarantee efficacy. This work provides a foundation for optimizing non-invasive light-based strategies in regenerative medicine and supports further translation towards SM tissue engineering applications. It, however, should be stated that future recommendations require a repetition of the same experiment in a three-dimensional environment to better modulate the in vivo environment and therefore assess whether results obtained will be maintained in the presence of a different and more realistic environmental cue for better regenerative medicine and tissue engineering translation.

## Figures and Tables

**Table 1:** Laser Parameters.

Parameters	Near-Infrared (NIR)	Green (G)
Light source	Diode	Diode
Wavelength (nm)	825	525
Emission	Continuous wave	Continuous wave
Power output (mW)	516	128
Power density (mW/cm^2^)	56.83	14,09
Fluence (J/cm^2^)	5/10	5/10
Exposure time (s)	87 and 175	354 and 709
Spot size (cm^2^)	9.62	9.62

**Table 2:** Normalization of scratch assay performed at Day 14 (Remaining scratch %).

Groups	0 h (%)	24 h (%)	48 h (%)	72 h (%)
	(Mean ± SD)	(Mean ± SD)	(Mean ± SD)	(Mean ± SD)
Control	100 ± 0.00	76.00 ± 10.58	76.67 ± 6.66	56.67 ± 7.77
525 nm 5 J/cm^2^	100 ± 0.00	93.00 ± 5.29 *	89.00 ± 5.57	84.67 ± 6.67 ****
825 nm 5 J/cm^2^	100 ± 0.00	91.00 ± 8.00 #	80.67 ± 8.08 ##	77.00 ± 7.00 ** ####
Combination 5 J/cm^2^	100 ± 0.00	84.33 ± 6.66	74.33 ± 11.06	63.00 ± 10.82
525 nm 10 J/cm^2^	100 ± 0.00	86.00 ± 5.29	78.00 ± 8.72	69.00 ± 9.64
825 nm 10 J/cm^2^	100 ± 0.00	74.33 ± 4.73 #	59.00 ± 2.00 * ##	48.67 ± 5.69 ####
Combination 10 J/cm^2^	100 ± 0.00	80.00 ± 3.46	66.67 ± 6.43	53.00 ± 4.36
Standard	100 ± 0.00	94.33 ± 4.93 *	86.67 ± 8.02	82.33 ± 7.23 ***

**Table 3:** Summary of key findings.

Assays	24 h	Day 7	Day 14	Overall Interpretation
Morphology	Uniform spindle-shaped ADSCs across all groups	Increased proliferation; 5 J/cm^2^ groups displayed denser cells, whilst 10 J/cm^2^ appeared sparser; standard group displayed fibroblast like morphology	Reduced cell density but maintained spindle SMC-like morphology; standard group cells appear unhealthy	PBM, particularly 5 J/cm^2^ supports SMC-like morphology; standard shows poor phenotype
ATP	Comparable to the control, combination 10 J/cm^2^ significantly increased	5 J/cm^2^ maintained (NIR) or increased (G and Combination) ATP; 10 J/cm^2^ decreased ATP significantly; standard displayed the highest ATP	Overall decline in ATP levels; 10 J/cm^2^ displayed significantly low ATP levels	Notable biphasic response with 5 J/cm^2^ promoting proliferation, whilst 10 J/cm^2^ suppresses metabolic activity
LDH cytotoxicity	Low across all groups; significant increase in G 5 and 10 J/cm^2^	Minimal cytotoxicity observed; decrease in cytotoxicity in the combination 5 J/cm^2^ group	All groups were low; 10 J/cm^2^ was significantly decreased compared to 5 J/cm^2^ counterpart	PBM is non-toxic; higher fluence showed reduced detectable membrane damage
MMP	Control group with the highest levels; no significant PBM effect	NIR 10 J/cm^2^ with significantly lower levels; other groups had slightly lower levels	PBM groups (G 5 and 10 J/cm^2^ and Combination 5 J/cm^2^) trended non-significantly higher than the control	Slightly lower yet comparable MMP levels over time, except with NIR 10 J/cm^2^ at midpoint showing inhibitory effects at higher fluence; PBM groups trended non-significantly higher at later stages, indicative of differentiation-driven energy demand
Collagen (ECM production)	Similar across groups; slight non-significant decrease in G 10 J/cm^2^, combination 5 J/cm^2^ and the standard	Non-significant increase across all groups; highest in G 5 J/cm^2^	Decreases (non-significantly) but remains higher in PBM groups; standard displays the lowest (non-significant collagen levels	5 J/cm^2^ enhances ECM production; supports differentiation
Migration	-	-	Increased migration in 10 J/cm^2^ groups	Higher fluence promotes a synthetic-like/migratory phenotype
MSC marker expression	Baseline ADSCs characterization and markers confirmed	-		Presence of MSC markers along with plasticity confirms ADSCs as MSCs
SMαA (Early marker)	Minimal expression across all groups	Upregulated in PBM groups, especially 5 J/cm^2^, with G 5 J/cm^2^ showing better organization; weaker and less defined but still evident in 10 J/cm^2^ groups	Control and 5 J/cm^2^ show strong staining; G 5 J/cm^2^ most organized; 10 J/cm^2^ showing weaker, delayed improvement	Early cytoskeletal organization occurs in all groups but is enhanced in 5 J/cm^2^, particularly greenlight
Desmin (Early marker)	Minimal expression across all groups	Upregulation of desmin begins; 5 J/cm^2^ groups are more structured; control shows moderate levels, while 10 J/cm^2^ shows weak staining	Dense but weakly stained desmin filament in the control group compared to 5 J/cm^2^ PBM groups; G 5 J/cm^2^ most defined compared to similar fluence groups; 10 J/cm^2^ groups present but weak	Desmin upregulation is fluence-dependent, with the strongest staining at lower fluence
Calponin (mid-stage marker)	Not evident across all groups	Increased expression in all PBM groups; more prominent in 5 J/cm^2^ compared to higher fluence; moderate expression in the control	Strong expression in control and 5 J/cm^2^ PBM groups, with G 5 J/cm^2^ showing the highest organization; weak expression in the 10 J/cm^2^ groups	5 J/cm^2^ PBM groups indicate progression toward a contractile-like phenotype, whilst higher fluence embodies a synthetic-like (dedifferentiated) phenotype at this stage
SMMHC (late marker)	Absent across all groups	5 J/cm^2^ groups show weak marker expression more evident than the control expression; 10 J/cm^2^ shows feeble expression; the standard shows no clear expression in the standard	Control and 5 J/cm^2^ groups show maturation into a contractile-like SMC phenotype as seen by strong expression of this late marker, more enhanced in 5 J/cm^2^ PBM groups	

## Data Availability

The original contributions presented in this study are included in the article. Further inquiries can be directed to the corresponding author.

## Abbreviations

The following abbreviations are used in this manuscript:

PBM	Photobiomodulation
SM	Smooth muscle
SMC	Smooth muscle cells
CCO	Cytochrome c oxidase
RLU	Relative light units
ATP	Adenosine triphosphate
LDH	Lactate dehydrogenase
MMP	Mitochondrial membrane potential
ADSCs	Adipose-derived stem cells
CD	Cluster of differentiation
SMMHC	Smooth muscle myosin heavy chain
SMAα	Smooth muscle actin alpha
IF	Immunofluorescence
NIR	Near infra-red
ECM	Extracellular matrix
RA	Retinoic acid
TGF-β	Transforming growth factor beta
MSC	Mesenchymal stem cells
PBS	Phosphate-buffered saline
FBS	Fetal Bovine serum
SMIM	Smooth muscle induction medium
DMEM	Dulbecco’s modified eagle medium
hTERT	Human telomerase reverse transcriptase
